# SARS-CoV-2 risk in household contacts of healthcare workers: a prospective cohort study

**DOI:** 10.1186/s13756-023-01300-5

**Published:** 2023-09-08

**Authors:** Philipp Kohler, Tamara Dörr, Andrée Friedl, Reto Stocker, Danielle Vuichard, Stefan P. Kuster, Christian R. Kahlert, the SURPRISE Study Group, Ulrike Besold, Elsbeth Betschon, Angela Brucher, Alexia Cusini, Andrée Friedl, Stephan Goppel, Fabian Grässli, Christian R. Kahlert, Joelle Keller, Simone Kessler, Philipp Kohler, Stefan P. Kuster, Onicio Leal, Eva Lemmenmeier, Allison McGeer, Dorette Meier Kleeb, Elisabeth Möller, J. Carsten Möller, Maja F. Müller, Vaxhid Musa, Manuela Ortner, Philip Rieder, Lorenz Risch, Markus Ruetti, Matthias Schlegel, Hans-Ruedi Schmid, Reto Stocker, Pietro Vernazza, Matthias von Kietzell, Danielle Vuichard-Gysin, Benedikt Wiggli

**Affiliations:** 1 Division of Infectious Diseases and Hospital Epidemiology, Cantonal Hospital St Gallen, St. Gallen, Switzerland; 2 Division of Infectious Diseases and Hospital Epidemiology, Cantonal Hospital Baden, Baden, Switzerland; 3 Hirslanden Clinic, Zurich, Switzerland; 4 Division of Infectious Diseases and Hospital Epidemiology, Thurgau Hospital Group, Muensterlingen, Switzerland; 5 Swiss National Centre for Infection Prevention (Swissnoso), Berne, Switzerland; 6 Department of Infectious Diseases and Hospital Epidemiology, Children’s Hospital of Eastern Switzerland, St. Gallen, Switzerland

**Keywords:** Healthcare workers, Household contacts, COVID-19, SARS-CoV-2, Spillover

## Abstract

**Background:**

Few studies have assessed whether the increased SARS-CoV-2 risk of healthcare workers (HCW) is carried on to their household contacts. Within a prospective HCW cohort, we assessed the SARS-CoV-2 risk of household contacts of HCW depending on the HCWs cumulative exposure to COVID-19 patients and identified factors influencing this association.

**Methods:**

HCW aged ≥ 16 years from nine Swiss healthcare networks participated. HCW without any household contacts were excluded. For HCW, cumulative patient exposure (number of COVID-19 patient contacts times average contact duration during a 12-month follow-up) was calculated. During follow-up, HCW reported SARS-CoV-2 nasopharyngeal swab results and positive swab results of their household contacts. We used multivariable logistic regression to identify variables associated with SARS-CoV-2 household positivity.

**Results:**

Of 2406 HCW, 466 (19%) reported ≥ 1 SARS-CoV-2 positive household. In multivariable analysis, patient exposure of HCW (adjusted OR [aOR] 1.08 per category, 95% CI 1.04–1.12), household size (aOR 1.53 per household member, 95% CI 1.35–1.73) and having children (aOR 0.70, 95% CI 0.53–0.94) remained associated with household positivity. Vaccinated HCW had a lower risk (aOR 0.54, 95% CI 0.38–0.77) of reporting a positive contact, as were those using respirator masks in contact with COVID-19 patients (aOR 0.65, 95% CI 0.49–0.86). Among vaccinated HCW, delayed first vaccination was associated with increased household SARS-CoV-2 positivity (aOR 1.14 per month, 95% CI 1.08–1.21).

**Conclusions:**

SARS-CoV-2 positivity in household contacts of HCW increases with higher cumulative COVID-19 patient exposure of HCWs. Measures reducing the SARS-CoV-2 risk in HCW might indirectly reduce the infection risk of their households.

**Supplementary Information:**

The online version contains supplementary material available at 10.1186/s13756-023-01300-5.

## Introduction

Healthcare workers (HCW) are at increased risk for acquiring SARS-CoV-2, particularly those caring for COVID-19 patients [1]. Previous data indicate that this risk gradually increases with higher cumulative exposure to COVID-19 patients [2]. This occupational risk has been shown to indirectly affect household contacts of HCW. Early on in the pandemic, family members of HCW had a twofold increased risk for hospitalization due to COVID-19 compared to the general population [3]. Nevertheless, few data exist on the extent of this suggested spillover effect and how it could be mitigated.

Within a prospective HCW cohort, we aimed to assess the SARS-CoV-2 risk of household contacts of HCW depending on the HCWs cumulative exposure to patients with COVID-19. Also, we sought to identify modifiable risk factors for SARS-CoV-2 positivity among contacts.

## Methods

The prospective multicentre cohort study, performed in nine healthcare networks in Switzerland, recruited volunteer HCW with and without patient exposure [4]. HCW ≥ 16 years were approached via institutional news tickers between June and September 2020. Upon electronic consent, HCW provided baseline information regarding their profession and household characteristics. For this analysis, HCW without any household contacts were excluded.

During a 12-month follow-up, HCW reported SARS-CoV-2 nasopharyngeal swab results and any positive SARS-CoV-2 swab among their household contacts. According to national public health recommendations, HCW and their household contacts were asked to undergo SARS-CoV-2 testing in case of compatible symptoms. HCW also reported any previous SARS-CoV-2 vaccination, whereas this information was not available from household contacts. SARS-CoV-2 swab results of HCW were validated as previously reported [5]. In September 2021, HCW reported the number of COVID-19 patient contacts (range 0–100) and average contact duration (range 1–60 min) during the past 12 months. Cumulative patient exposure (number of contacts times the average contact duration) was grouped into eight categories defined by powers of two, as described previously [2]. At baseline, in January and September 2021, participating HCW were screened for anti-nucleocapsid (anti-N) antibodies. SARS-CoV-2 positivity in HCW was defined as self-reported positive swab and/or anti-N seroconversion.

The outcomes were percentage of SARS-CoV-2 positivity among households and HCW (for analyses of grouped data), and having at least one positive SARS-CoV-2 swab (vs. none) among any household contact during the study period (for analyses of individual data). We used linear regression analysis to determine the association of household (and HCW) SARS-CoV-2 positivity across categories of cumulative patient contact of index HCWs. For the individual level analyses, we used logistic regression and analyzed variables with known or presumed association with SARS-CoV-2 household positivity, including having pets [6]. For the multivariable analysis, we included all a priori defined variables (model 1) (Additional file 1: Table S1). To assess the impact of SARS-CoV-2 infection of the index HCW on household positivity, a second model (model 2) was fitted adding this variable to model 1. Furthermore, we excluded unvaccinated HCW considering that unvaccinated HCW might be more likely to co-habit with unvaccinated households; instead, we introduced the variable “Month of first SARS-CoV-2 vaccination” (i.e. December 2020 defined as month 1 up to October 2021 or later defined as month 11) in the model, to see whether delayed vaccination had an impact on household positivity (model 3). Odds ratios (OR) and corresponding 95% were calculated; *p*-values of < 0.05 were considered significant. R statistical software Version 3.6.1 was used for statistical analysis. We followed the STROBE reporting guideline for observational studies.

## Results

Upon recruitment in August 2020, 4185 individuals reported having at least one household contact. Of these, 2406 (57%) HCW answered the questionnaire in September 2021; 78% were female and median age was 43 years (range 18–68). Including the index HCW, the median household size was 3 (range 2–10 people). Of 2406 participants, 466 (19%) reported ≥ 1 SARS-CoV-2 positive household. Baseline characteristics were similar between HCW with and without positive household contacts (Additional file 1: Table S2).

**Fig. 1:**
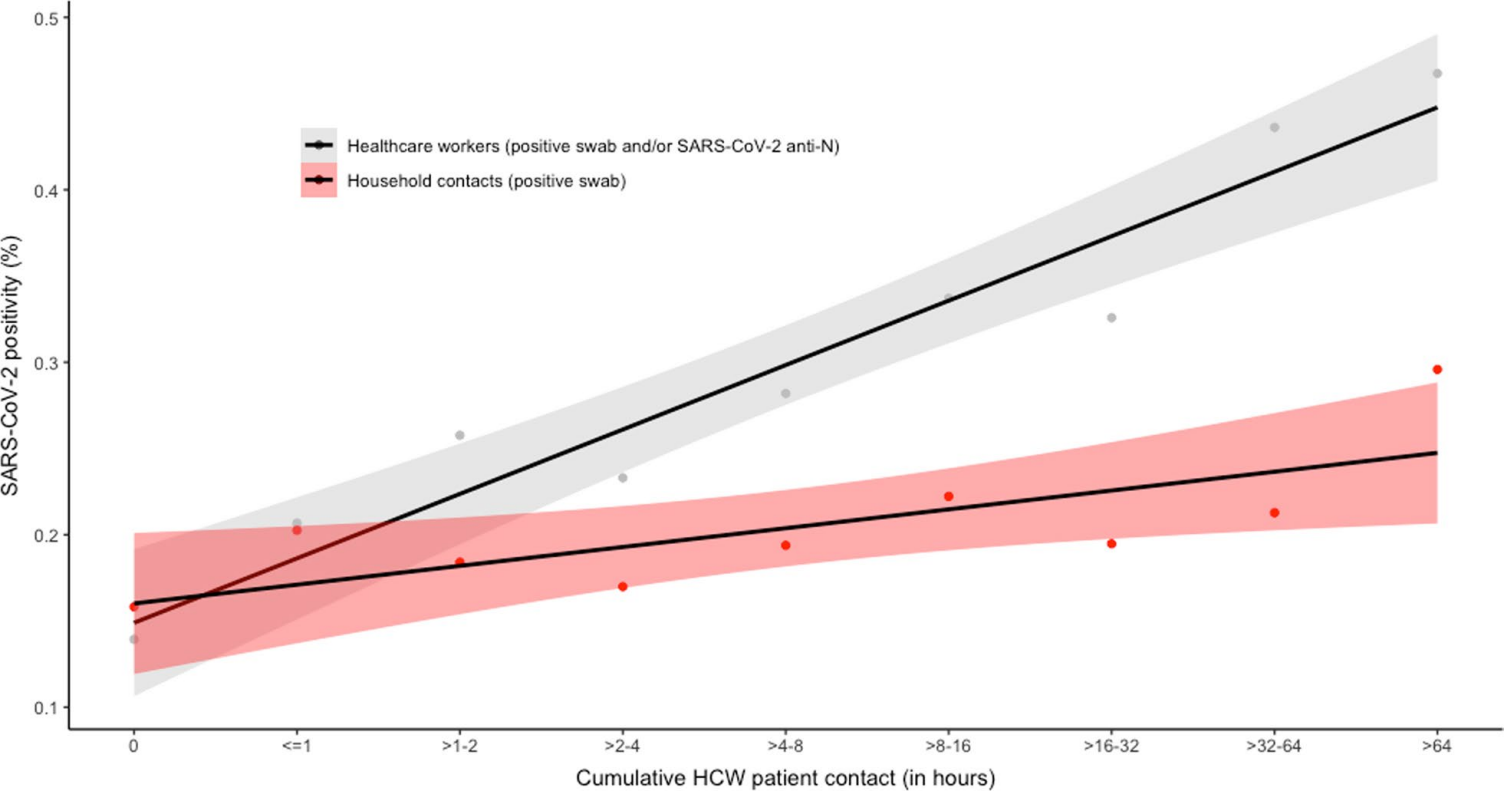
SARS-CoV-2-positivity in household contacts and in corresponding index healthcare workers by cumulative patient exposure. SARS-CoV-2 positivity in household contacts (red) and in corresponding index healthcare workers (grey), depending on cumulative patient exposure of healthcare workers. Black lines show graphs of linear regression analysis; shaded areas correspond to 95% confidence intervals

Between HCW without patient contact and those with highest (> 64 h) cumulative patient exposure, SARS-CoV-2 positivity for the respective household contacts increased from 16 to 30% (+ 1.0% percentage points per category, *p* < 0.001); positivity among HCW increased from 14 to 47% (+ 3.7%, *p* < 0.001) (Fig. 1, Additional file 1: Fig. S1).

In model 1, increasing patient exposure remained associated with household positivity (adjusted OR [aOR] 1.08 per category, 95% CI 1.04–1.12, *p* < 0.001). Household size (aOR 1.53 per additional household member, 95% CI 1.35–1.73, *p* < 0.001) and having children in the same household (aOR 0.70, 95% CI 0.53–0.94, *p* = 0.02) were also associated with the outcome. Vaccinated HCW had a lower risk (aOR 0.54, 95% CI 0.38–0.77, *p* < 0.001) of reporting a positive contact, as were those using respirator masks in contact with COVID-19 patients (aOR 0.65, 95% CI 0.49–0.86, *p* = 0.003). In model 2, SARS-CoV-2 positivity of HCW (aOR 10.6, 95% CI 8.3–13.7, *p* < 0.001) and household size (aOR 1.61, 95% CI 1.40–1.85, *p* < 0.001) were the only variables associated with the outcome, suggesting SARS-CoV-2 positivity in HCW to be an important mediator variable for the associations seen in model 1. In model 3, with every additional month of delayed first vaccination, the risk of SARS-CoV-2 in household increased significantly (aOR 1.14, 95% CI 1.08–1.21); estimates of the other co-variables were similar as in the other models (Table 1).

**Table 1:** Results of logistic regression regarding risk of having at least one positive household contact

	Univariable analysis N = 2406		Multivariable analysis (model 1), N = 2308		Multivariable analysis (model 2*), N = 2308		Multivariable analysis (model 3*), N = 1681	
	OR (95% CI)	*p*-value	OR (95% CI)	*p*-value	OR (95% CI)	p-value	OR (95% CI)	*p*-value
*HCW characteristics*								
Age (years), median (interquartile range)	0.99 (0.98–1.00)	**0.02**	0.99 (0.98–1.00)	0.06	0.99 (0.98–1.00)	0.20	0.99 (0.98–1.01)	0.45
Body mass index, median (range)	1.00 (0.97–1.02)	0.79	1.00 (0.98–1.03)	0.84	1.00 (0.98–1.03)	0.79	0.99 (0.96–1.03)	0.73
Male gender	1.05 (0.83–1.34)	0.68	1.16 (0.87–1.55)	0.33	1.26 (0.91–1.74)	0.17	1.25 (0.88–1.78)	0.22
Active smoker (vs. never/former)	0.75 (0.54–1.04)	0.09	0.77 (0.55–1.08)	0.13	0.93 (0.64–1.36)	0.72	0.86 (0.57–1.29)	0.46
At least one comorbidity	1.08 (0.88–1.32)	0.47	1.20 (0.97–1.49)	0.09	1.22 (0.96–1.55)	0.10	1.38 (1.06–1.79)	**0.02**
*HCW related factors*								
Cumulative patient contact in hours (OR per category)	1.07 (1.03–1.11)	** < 0.001**	1.08 (1.04–1.12)	** < 0.001**	0.96 (0.91–1.01)	0.08	1.11 (1.05–1.17)	** < 0.001**
Always respirator (vs. surgical/mixed mask use)	0.62 (0.47–0.82)	** < 0.001**	0.65 (0.49–0.86)	**0.003**	0.88 (0.64–1.21)	0.44	0.78 (0.55–1.10)	0.15
Working ≥ 80% FTE	0.88 (0.72–1.07)	0.20	0.91 (0.70–1.19)	0.51	0.83 (0.61–1.13)	0.23	0.80 (0.57–1.11)	0.17
Working in intensive care	1.10 (0.77–1.57)	0.59	1.09 (0.74–1.61)	0.66	1.32 (0.86–2.04)	0.20	1.13 (0.72–1.77)	0.60
SARS-CoV-2 vaccination	0.56 (0.43–0.72)	** < 0.001**	0.54 (0.38–0.77)	** < 0.001**	0.70 (0.47–1.04)	0.08	NA	
SARS-CoV-2 positivity of index HCW			NA		10.6 (8.3–13.7)	** < 0.001**	NA	
Month of SARS-CoV-2 vaccination	1.13 (1.07–1.19)		NA		NA		1.14 (1.08–1.21)	** < 0.001**
*Household related factors*								
Household size (per additional person)	1.43 (1.30–4.57)	** < 0.001**	1.53 (1.35–1.73)	** < 0.001**	1.61 (1.40–1.85)	** < 0.001**	1.55 (1.32–1.81)	** < 0.001**
Always wearing a mask outside work	1.25 (0.93–1.68)	0.15	1.22 (0.83–1.78)	0.32	0.99 (0.65–1.52)	0.97	1.15 (0.72–1.82)	0.56
At least one household contact with SARS-CoV-2 vaccination	0.79 (0.61–1.01)	0.06	1.12 (0.80–1.59)	0.51	1.15 (0.78–1.68)	0.48	1.47 (0.84–2.58)	0.17
Children in household	1.37 (1.12–1.68)	**0.003**	0.70 (0.53–0.94)	**0.02**	0.75 (0.55–1.04)	0.09	0.65 (0.46–0.93)	**0.02**
Pet in household	1.06 (0.86–1.30)	0.61	0.96 (0.78–1.19)	0.73	1.03 (0.81–1.31)	0.80	1.06 (0.81–1.38)	0.68

OR, Odds Ratio; CI, Confidence Interval; HCW, Healthcare worker; FTE, Full Time Equivalent

*Multivariable model 2 is identical to model 1, but additionally includes the variable “SARS-CoV-2-positivity of index healthcare worker”. Model 3 is identical to model 1, but unvaccinated healthcare workers are excluded and the variable “Month of first SARS-CoV-2 vaccination” is newly included

*P*-values with a significance level of < 0.05 are shown in bold

## Discussion

In this prospective cohort, SARS-CoV-2 positivity in household contacts of participating HCW increased with higher cumulative COVID-19 patient exposure of HCWs. The spillover effect of occupational SARS-CoV-2 risk was reduced in vaccinated HCW and those using respirator masks. Strengths of the study include the large sample size of over 2400 HCW and the repetitive serology testing of HCWs.

This study shows an intriguing dose-dependence effect of COVID-19 exposure in HCW and SARS-CoV-2 positivity in their households, mediated by SARS-CoV-2 infection in HCW with postulated subsequent transmission to their contacts. Few other studies have previously analyzed the COVID-19 risk in household contacts of HCW. In a large cohort from Scotland, household members of patient-facing HCW were at increased risk of being hospitalized with COVID-19, whereas hospitalization risk of households from non-patient-facing HCW was similar to the community population [3]. Although the directionality of SARS-CoV-2 transmission cannot be definitively assessed, the dose–response effect described in our data strongly supports the hypothesis of HCW putting their families at increased risk for SARS-CoV-2 acquisition.

We also identified two potentially modifiable factors, which were independently associated with reduced risk of reporting a SARS-CoV-2 positive household contact. First, HCW vaccination was associated with reduced risk; the effect size in our study (aOR 0.54) is in line with data from a study on over 265,000 HCW from Finland, which found an indirect vaccine effectiveness of 39% (for unvaccinated household contacts) [7]. Similarly, in a population-based study from England, the likelihood of SARS-CoV-2 intra-household transmission was reduced by 40–50% after at least one vaccine dose [8]. Supporting a causal relationship, delayed vaccination of HCW was associated with increased SARS-CoV-2 risk in their households in our sensitivity analysis. Second, wearing respirators consistently in contact with COVID-19 patients was also associated with decreased risk, with an aOR of 0.65. However, in contrast to SARS-CoV-2 vaccination, a recently published randomized trials showed no benefit of respirator over surgical masks in preventing SARS-CoV-2 infection [9]. Given our observational study design, no conclusions should be drawn from this finding, which might also be at least partly explained by residual confounding.

Importantly, we did not capture asymptomatic infections of households, which may account for more than one third of infections [10]. This limitation might underestimate the effect observed in our study. The study was performed before emergence of the Omicron variant, which limits the generalizability of our findings. In fact, data suggest that the secondary attack rate within households is higher with the Omicron variant compared to Delta [11]. Also, we did not account for the individual vaccination status of household contacts. Indeed, unvaccinated HCW might be more likely to co-habit with unvaccinated households; yet, excluding unvaccinated HCW did not significantly alter our results. Another limitation is that no systematic SARS-CoV-2 testing was performed in HCW and their households, and that we relied on self-reporting of SARS-CoV-2 positive results. However, based on our experience in validating self-reported swab results of HCW [5], we think that data on self-reported swab results are reliable. Furthermore, residual confounding prevents us from inferring any causal effects for the observed associations.

## Conclusion

These data suggest that household contacts of HCW are at increased risk of acquiring SARS-CoV-2, depending on the intensity of the HCWs patient contact. Measures reducing the risk of SARS-CoV-2 positivity in HCW may indirectly reduce the COVID-19 risks among their households.

### Supplementary Information

**Additional file 1. Table S1**. A priori defined co-variables, including definitions, answer levels and questionnaire. **Table S2**. Baseline characteristics of healthcare workers without and with at least one SARS-CoV-2 positive household contact. **Figure S1.** Percentage and number of households with at least one infected individual (y-axis) by cumulative healthcare worker patient contact in hours (x-axis).

## Data Availability

The datasets used and/or analysed during the current study are available from the corresponding author on reasonable request.

## Abbreviations

Anti-N: Anti-nucleocapsid
Anti-S: Anti-spike
aOR: Adjusted odds ratio
CI: Confidence interval
COVID-19: Coronavirus disease 2019
HCW: Healthcare workers
OR: Odds ratio
SARS-CoV-2: Severe acute respiratory syndrome coronavirus type 2
