# Supplementary material for: SARS-CoV-2 risk in household contacts of healthcare workers: a prospective cohort study
Source: Antimicrob Resist Infect Control. 2023 Sep 8;12:98. doi: 10.1186/s13756-023-01300-5 (PMC10492321; doi:10.1186/s13756-023-01300-5)
Supplement: Supplementary file 1 — Additional file 1. Table S1. A priori defined co-variables, including definitions, answer levels and questionnaire. Table S2. Baseline characteristics of healthcare workers without and with at least one SARS-CoV-2 positive household contact. Figure S1. Percentage and number of households with at least one infected individual (y-axis) by cumulative healthcare worker patient contact in hours (x-axis). [file 13756_2023_1300_MOESM1_ESM.docx]

**SARS-CoV-2 risk in household contacts of healthcare workers – a prospective cohort study**

**SUPPLEMENTARY TABLES**

**Table S1.** *A priori* defined co-variables, including definitions, answer levels and questionnaire.

| **Variable name (unit)** | **Definition** | **Levels** | **Questionnaire** |
| --- | --- | --- | --- |
| **Anthropometrics/baseline health** | | | |
| Age (years) | Age at time of last serology | Number | Follow-up |
| Body mass index (kg/m2) | BMI at time of study entry | Number | Baseline |
| Sex | According to participants choice | Female; Male (Ref) | Baseline |
| Smoking status | Smoking status at time of study entry | Active; Never/former (Ref) | Baseline |
| Comorbidities | Presence of any of the following: arterial hypertension, diabetes, cancer, pulmonary disease, rheumatologic disease, other | Yes; No (Ref) | Baseline |
| **Healthcare worker related factors** | | | |
| Fulltime work ≥80% | Fulltime equivalent ≥80% at study entry | Yes; No (Ref) | Baseline |
| Works in intensive care | Works in intensive care at study entry | Yes; No (Ref) | Baseline |
| Cumulative patient exposure (in hours, h) | (Number of COVID-19 patients which participant was exposed to since beginning of the study) x (average duration of patient contact) | Categorized by power of two (i.e. 1h, >1-2h; >2-4h; >4-8h; >8-16h; >16-32h; >32-64h; >64h); no patient contact (Ref) | Follow-up |
| Mask type | Mask type preferentially used during COVID-19 patient contact during study period (outside of aerosol-generating procedures) | Always respirator; mixed/mostly surgical masks/no COVID-19 patient contact (Ref) | Follow-up |
| SARS-CoV-2 vaccination in HCW | Having had ≥1 SARS-CoV-2 vaccination | Yes; No (Ref) | Follow-up |
| Months of first SARS-CoV-2 vaccine | Month of first SARS-CoV-2 vaccine according to self-reported date | 1 (December 2020) through 11 (October 2021 or later) | Follow-up |
| Always mask outside of work | Wearing mostly mask in public locations | Yes; No (Ref) | Follow-up |
| **Household related factors** | | | |
| **Positive household contact (main outcome)** | **At least one household contact with positive SARS-CoV-2 swab at any time point** | **Yes; No (Ref)** | **Follow-up** |
| Household size | Number of additional people living in household | (0, excluded), 1,2,3, 4 or more | Baseline |
| Children in household | At least one child (<18 y) living in household | Yes; No (Ref) | Baseline |
| Pet | At least one pet in household | Yes; No (Ref) | Baseline |
| SARS-CoV-2 vaccination in household | At least one household contact with SARS-CoV-2 vaccination during study period | Yes; No (Ref) | Follow-up |

Kg, Kilogram; m, Meter; Ref, Reference; y, years

**Table S2**. Baseline characteristics of healthcare workers without and with at least one SARS-CoV-2 positive household contact.

|  | No positive household (n=1940) | Positive household (n=466) |
| --- | --- | --- |
|  | n (%)^*^ | n (%)^*^ |
| Baseline |  |  |
| Age (years), median (interquartile range) | 39 (30-49) | 40 (32-49) |
| Body mass index, median (range) | 24 (21-26) | 24 (21-26) |
| Male gender | 428 (22.1) | 107 (23.0) |
| Active smoker (vs. never/former) | 252 (13.0) | 47 (10.1) |
| At least one comorbidity | 764 (39.4) | 192 (41.2) |

^*^ if not stated otherwise

Abbreviations: SARS-CoV-2, Severe Acute Respiratory Syndrome Coronavirus 2

**Figure S1.** Percentage and number of households with at least one infected individual (y-axis)

by cumulative healthcare worker patient contact in hours (x-axis).

**
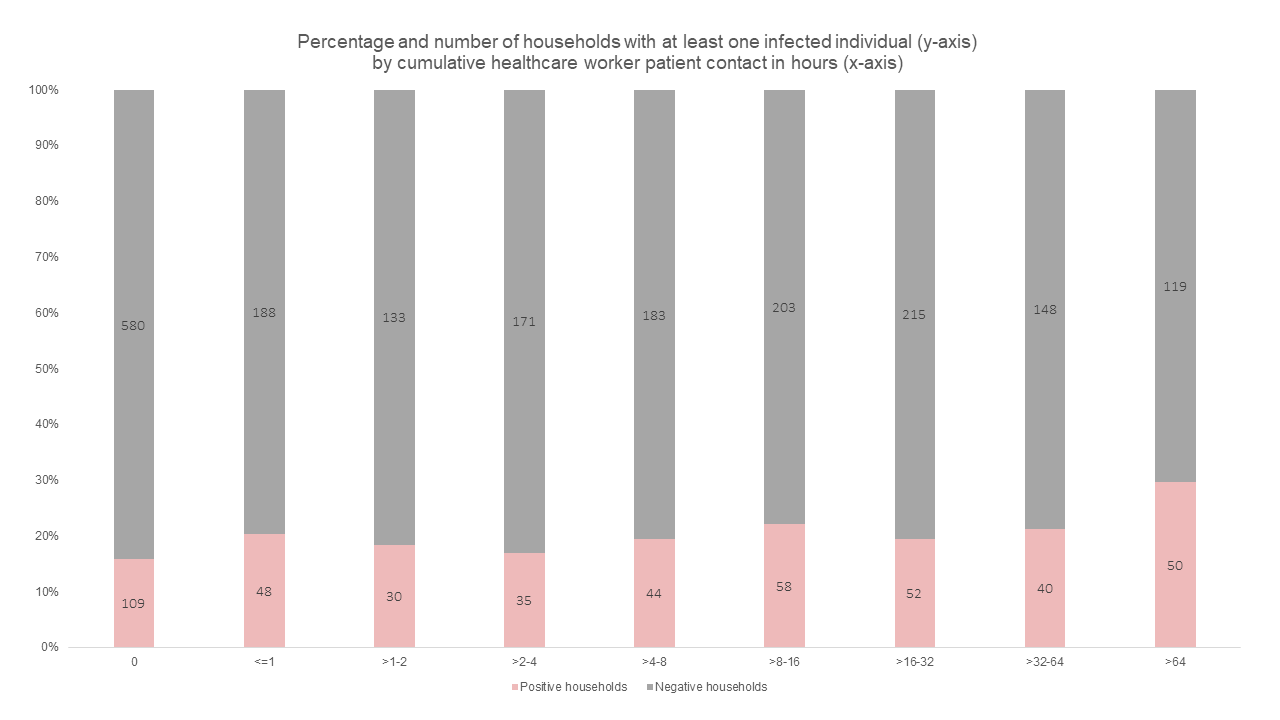
**
